# Supplementary figures and images for: Analysis of patients preferences in type 2 diabetes mellitus second-line drug treatment: A discrete choice experiment
Source: PLoS One. 2025 Sep 15;20(9):e0329743. doi: 10.1371/journal.pone.0329743 (PMC12435682; doi:10.1371/journal.pone.0329743)

*S1 Figure – PRISMA Diagram*

*
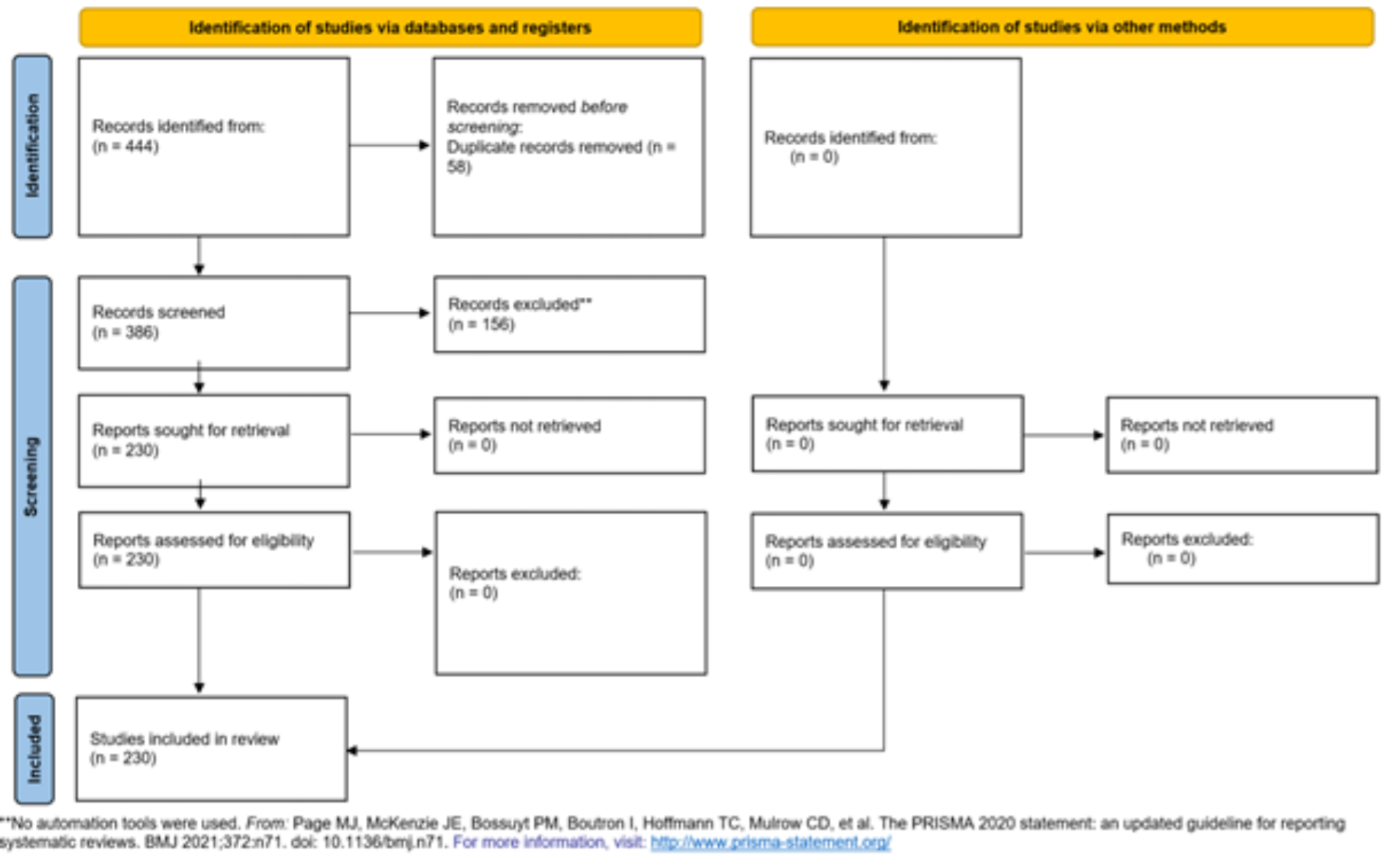
*

Supplement: S1 Fig — (DOCX) [file pone.0329743.s001.docx]

*S2 Figure – Decision model*


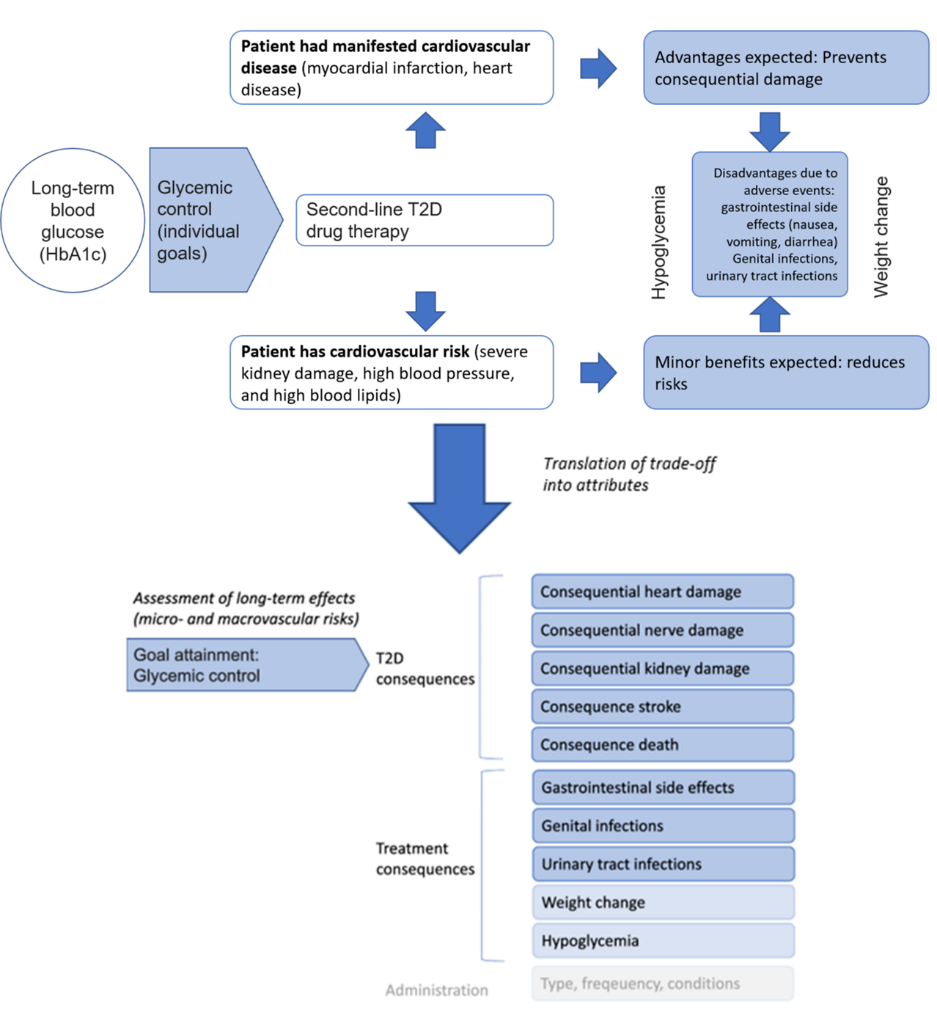

Supplement: S2 Fig — (DOCX) [file pone.0329743.s002.docx]

*S3 Figure – Choice Task, example*


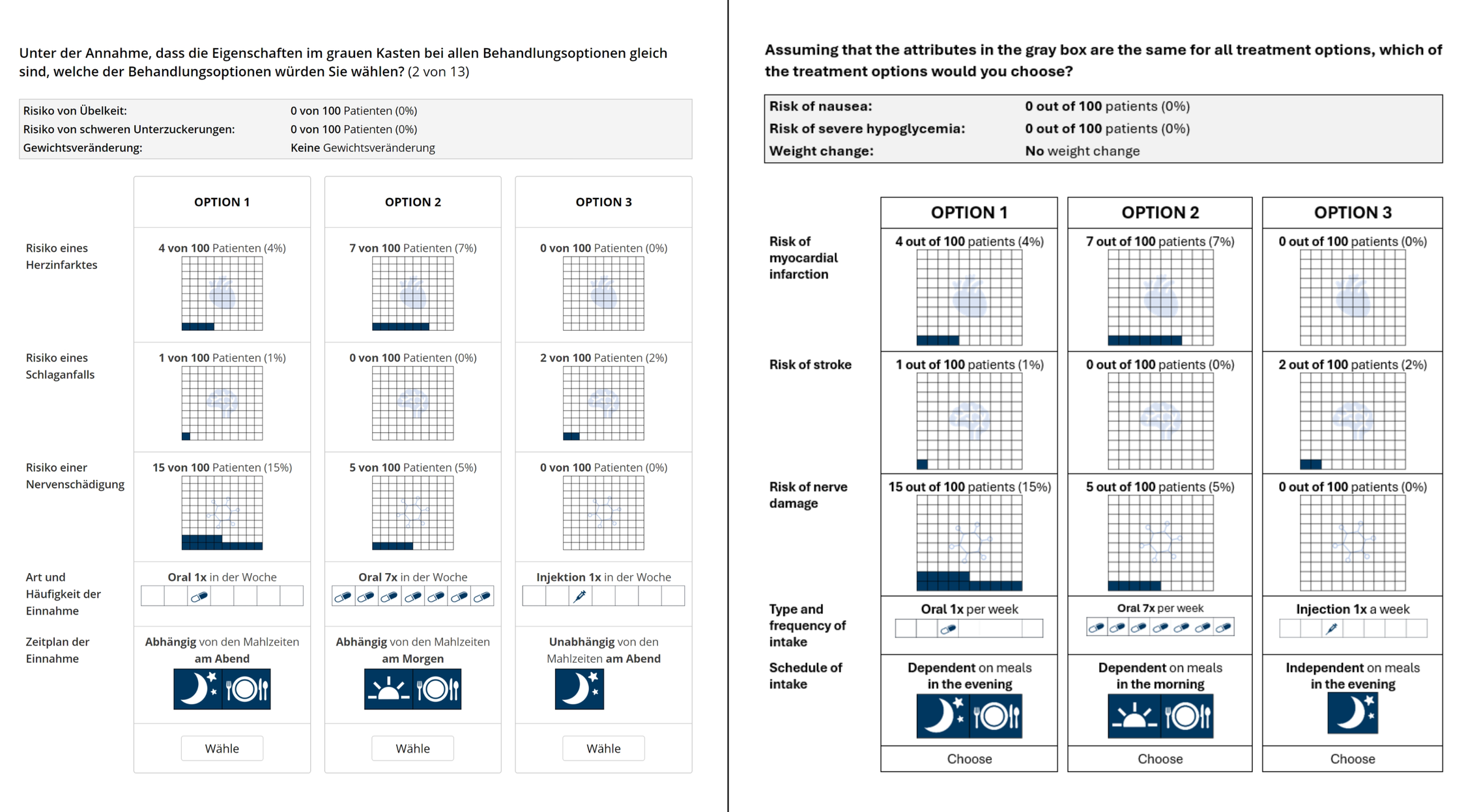

**Original German version and translated version.*

Supplement: S3 Fig — (DOCX) [file pone.0329743.s003.docx]
