# Supplementary material for: Analysis of patients preferences in type 2 diabetes mellitus second-line drug treatment: A discrete choice experiment
Source: PLoS One. 2025 Sep 15;20(9):e0329743. doi: 10.1371/journal.pone.0329743 (PMC12435682; doi:10.1371/journal.pone.0329743)
Supplement: S1 Table — (DOCX) [file pone.0329743.s004.docx]

*S1 Table – Attribute list*

| T2D consequences  (disease-related effects)  **N = 13** | Treatment consequences  (therapy-related effects)  **N = 7** | Administration  (mode of application for the pharmaceutical product)  **N = 4** |
| --- | --- | --- |
| *Risk of myocardial infarction* | *Weight change in kg* | *Type of application* |
| *Risk of hospitalization due to heart failure (heart failure-related hospitalization)* | *Risk of low blood sugar (hypoglycemia)* | *Frequency of use (dosage)* |
| *Risk of cardiovascular death* | *Vomitting* | *Timing of application* |
| *Risk of stroke* | *Nausea* | *Complexity of application* |
| *Blood pressure change* | *Diarrhea* |  |
| *Change in heart rate (pulse)* | *Urinary tract infection* |  |
| *Change in cholesterol levels* | *Genital infection* |  |
| *Risk of death (all-cause mortality)* |  |  |
| *Change in long-term blood glucose levels*  */ HbA1c change in %* |  |  |
| *Risk of kidney disease (nephropathy)* |  |  |
| *Risk of nerve damage (neuropathy)* |  |  |
| *Risk of amputations* |  |  |
| *Risk of eye damage (retinopathy)* |  |  |
