# Supplementary material for: Analysis of patients preferences in type 2 diabetes mellitus second-line drug treatment: A discrete choice experiment
Source: PLoS One. 2025 Sep 15;20(9):e0329743. doi: 10.1371/journal.pone.0329743 (PMC12435682; doi:10.1371/journal.pone.0329743)
Supplement: S2 Table — (DOCX) [file pone.0329743.s005.docx]

*S2 Table – Descriptive Framework*

| Attribute | Level 1 | Level 2 | Level 3 | Level 4 |
| --- | --- | --- | --- | --- |
| Risk of myocardial infarction | 0 out of 100 (0%) | 2 out of 100 (2%) | 4 out of 100 (4%) | 7 out of 100 (7%) |
|  | 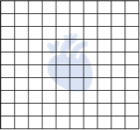 | 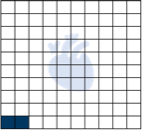 | 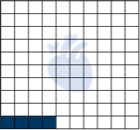 | 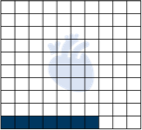 |
| Type 2 diabetes increases the risk of a myocardial infarction, but this can be reduced with medication. Myocardial infarctions require medical intervention and lead to permanent damage to the heart muscle. The risk of a myocardial infarction is illustrated by its occurrence in a certain number of patients. | | | | |
| Risk of stroke | 0 out of 100 (0%) | 1 out of 100 (1%) | 2 out of 100 (2%) | 4 out of 100 (4%) |
|  | 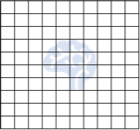 | 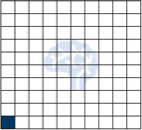 | 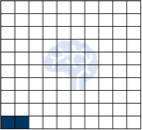 | 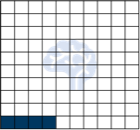 |
| Type 2 diabetes heightens the risk of strokes, but drug treatment can mitigate this risk. Strokes may impair physical and mental functions, including perception and emotions. The risk of a stroke is illustrated by its occurrence in a certain number of patients. | | | | |
| Risk of neuropathy | 0 out of 100 (0%) | 5 out of 100 (5%) | 10 out of 100 (10%) | 15 out of 100 (15%) |
|  | 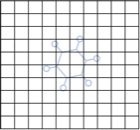 | 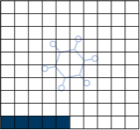 | 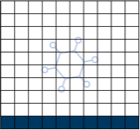 | 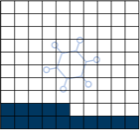 |
| Type 2 diabetes can increase the risk of nerve damage, which can be reduced with medication. Nerve damage is associated with permanent impairments such as sensory disturbances. This risk increases with the duration of the disease. Risk of nerve damage is illustrated by its occurrence in a certain number of patients. | | | | |
| Risk of nausea | 0 out of 100 (0%) | 10 out of 100 (10%) | 30 out of 100 (30%) | 50 out of 100 (50%) |
|  | 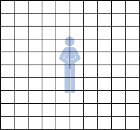 | 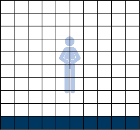 | 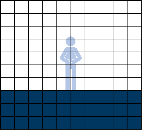 | 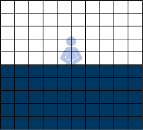 |
| Nausea can occur as a side effect of drug treatment for type 2 diabetes. In the DCE, nausea is assumed to be moderate, occurring regularly and very frequently (once or several times a week). The risk of nausea is illustrated by its occurrence in a certain number of patients. | | | | |
| Risk of severe hypoglycemia | 0 out of 100 (0%) | 1 out of 100 (1%) | 2 out of 100 (2%) | 4 out of 100 (4%) |
|  | 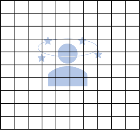 | 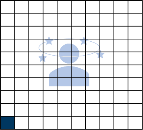 | 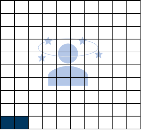 | 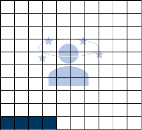 |
| Hypoglycemia, a possible side effect of drug treatment for type 2 diabetes, can have serious consequences such as seizures or respiratory disorders. Severe hypoglycemia poses a life-threatening risk and requires immediate medical attention. It usually occurs once or several times a year. Risk of severe hypoglycemia is illustrated by its occurrence in a certain number of patients. | | | | |
| Weight change | -6kg | -2kg | +2kg | +6kg |
|  | 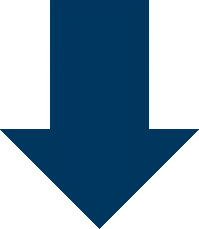 | 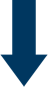 | 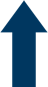 | 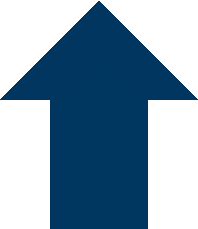 |
| Weight changes can be caused by the drug treatment of type 2 diabetes. This can result in increasing or decreasing weight. | | | | |
| Mode and frequency of intake | Oral 1x per week | Oral 7x per week | Injection 1x per week | Injection 7x per week |
|  | 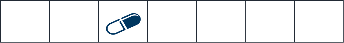 | 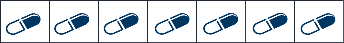 | 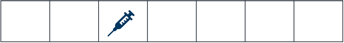 | 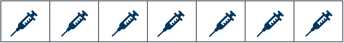 |
| The type and frequency of administration vary when treating type 2 diabetes with medication. A diabetes medication can be taken orally or injected. It can be taken once or several times a week. | | | | |
| Schedule of intake | Independent from meal at the morning | Dependent from meal at the morning | Independent from meal at the evening | Dependent from meal at the evening |
|  | 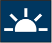 | 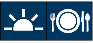 | 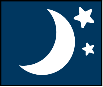 | 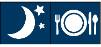 |
| The schedule for taking medication for type 2 diabetes varies. There are medications that must be taken depending on or independently of meals. Medication can be taken in the morning or in the evening. | | | | |

*The descriptive framework was translated from German into English. However, it is important to note that the study participants only read the German version.*
