# Supplementary material for: Analysis of patients preferences in type 2 diabetes mellitus second-line drug treatment: A discrete choice experiment
Source: PLoS One. 2025 Sep 15;20(9):e0329743. doi: 10.1371/journal.pone.0329743 (PMC12435682; doi:10.1371/journal.pone.0329743)
Supplement: S3 Table — (DOCX) [file pone.0329743.s006.docx]

*S3 Table – CL by study populations*

|  |  | **Population 1**  **(N=292)** |  |  | | | | **Population 2**  **(N=291)** |  |  | | | |
| --- | --- | --- | --- | --- | --- | --- | --- | --- | --- | --- | --- | --- | --- |
| **Attributes** | **Levels** | **Mean** | **se** | **p** | | **95% CI** | | **Mean** | **se** | **p** | | **95% CI** | |
| Risk of myocardial infarction | 0 out of 100 patients (0%) | 0.670 | 0.057 | 0.000 | 0.557 | | 0.782 | 0.762 | 0.059 | 0.000 | 0.647 | | 0.877 |
|  | 2 out of 100 patients (2%) | 0.177 | 0.059 | 0.003 | 0.061 | | 0.293 | 0.183 | 0.060 | 0.002 | 0.066 | | 0.299 |
|  | 4 out of 100 patients (4%) | -0.163 | 0.060 | 0.007 | -0.281 | | -0.045 | -0.324 | 0.062 | 0.000 | -0.447 | | -0.202 |
|  | 7 out of 100 patients (7%) | -0.684 | 0.065 | 0.000 | -0.811 | | -0.556 | -0.620 | 0.066 | 0.000 | -0.749 | | -0.490 |
| Risk of stroke | 0 out of 100 patients (0%) | 0.524 | 0.058 | 0.000 | 0.410 | | 0.638 | 0.578 | 0.060 | 0.000 | 0.461 | | 0.695 |
|  | 1 out of 100 patients (1%) | 0.113 | 0.059 | 0.058 | -0.004 | | 0.229 | 0.198 | 0.061 | 0.001 | 0.079 | | 0.318 |
|  | 2 out of 100 patients (2%) | -0.093 | 0.059 | 0.116 | -0.208 | | 0.023 | -0.222 | 0.062 | 0.000 | -0.344 | | -0.101 |
|  | 4 out of 100 patients (4%) | -0.544 | 0.065 | 0.000 | -0.671 | | -0.417 | -0.554 | 0.065 | 0.000 | -0.682 | | -0.426 |
| Risk of nerve damage | 0 out of 100 patients (0%) | 1.375 | 0.061 | 0.000 | 1.255 | | 1.494 | 1.610 | 0.065 | 0.000 | 1.482 | | 1.737 |
|  | 5 out of 100 patients (5%) | 0.277 | 0.059 | 0.000 | 0.161 | | 0.392 | 0.303 | 0.062 | 0.000 | 0.182 | | 0.425 |
|  | 10 out of 100 patients (10%) | -0.211 | 0.062 | 0.001 | -0.331 | | -0.090 | -0.171 | 0.063 | 0.007 | -0.294 | | -0.048 |
|  | 15 out of 100 patients (15%) | -1.441 | 0.081 | 0.000 | -1.600 | | -1.281 | -1.742 | 0.090 | 0.000 | -1.918 | | -1.567 |
| Risk of nausea | 0 out of 100 patients (0%) | 2.433 | 0.079 | 0.000 | 2.279 | | 2.587 | 2.501 | 0.080 | 0.000 | 2.345 | | 2.657 |
|  | 10 out of 100 patients (10%) | 0.995 | 0.066 | 0.000 | 0.866 | | 1.125 | 0.966 | 0.064 | 0.000 | 0.840 | | 1.092 |
|  | 30 out of 100 patients (30%) | -1.089 | 0.088 | 0.000 | -1.262 | | -0.917 | -1.342 | 0.089 | 0.000 | -1.516 | | -1.168 |
|  | 50 out of 100 patients (50%) | -2.339 | 0.124 | 0.000 | -2.583 | | -2.095 | -2.125 | 0.110 | 0.000 | -2.341 | | -1.908 |
| Risk of severe hypoglycemia | 0 out of 100 patients (0%) | 0.555 | 0.056 | 0.000 | 0.444 | | 0.665 | 0.609 | 0.059 | 0.000 | 0.494 | | 0.724 |
|  | 1 out of 100 patients (1%) | 0.146 | 0.058 | 0.012 | 0.032 | | 0.260 | 0.091 | 0.059 | 0.124 | -0.025 | | 0.208 |
|  | 2 out of 100 patients (2%) | -0.219 | 0.062 | 0.000 | -0.340 | | -0.098 | -0.147 | 0.061 | 0.016 | -0.267 | | -0.027 |
|  | 4 out of 100 patients (4%) | -0.482 | 0.064 | 0.000 | -0.608 | | -0.356 | -0.554 | 0.067 | 0.000 | -0.684 | | -0.423 |
| Weight change | Decrease of -6kg | 0.790 | 0.059 | 0.000 | 0.673 | | 0.906 | 1.183 | 0.063 | 0.000 | 1.060 | | 1.307 |
|  | Decrease of -2kg | 0.998 | 0.058 | 0.000 | 0.885 | | 1.112 | 1.080 | 0.061 | 0.000 | 0.961 | | 1.198 |
|  | Increase of +2kg | -0.377 | 0.064 | 0.000 | -0.504 | | -0.251 | -0.510 | 0.069 | 0.000 | -0.645 | | -0.375 |
|  | Increase of +6kg | -1.411 | 0.080 | 0.000 | -1.567 | | -1.254 | -1.753 | 0.091 | 0.000 | -1.930 | | -1.576 |
| Type and frequency of intake | Oral 1x per week | 0.626 | 0.042 | 0.000 | 0.543 | | 0.710 | 0.538 | 0.044 | 0.000 | 0.452 | | 0.624 |
|  | Oral 7 times a week | -0.149 | 0.044 | 0.001 | -0.236 | | -0.062 | -0.220 | 0.046 | 0.000 | -0.311 | | -0.130 |
|  | Injection 1x a week | 0.362 | 0.043 | 0.000 | 0.278 | | 0.446 | 0.347 | 0.044 | 0.000 | 0.261 | | 0.432 |
|  | Injection 7x a week | -0.840 | 0.050 | 0.000 | -0.938 | | -0.742 | -0.665 | 0.049 | 0.000 | -0.761 | | -0.568 |
| Schedule of intake | Independent of meals in the morning | 0.044 | 0.044 | 0.314 | -0.042 | | 0.130 | 0.062 | 0.044 | 0.160 | -0.025 | | 0.149 |
|  | Dependent on meals in the morning | -0.089 | 0.044 | 0.044 | -0.175 | | -0.002 | -0.140 | 0.045 | 0.002 | -0.229 | | -0.051 |
|  | Independent of meals in the evening | 0.022 | 0.043 | 0.607 | -0.063 | | 0.107 | 0.052 | 0.044 | 0.243 | -0.035 | | 0.138 |
|  | Dependent on meals in the evening | 0.022 | 0.043 | 0.603 | -0.062 | | 0.106 | 0.027 | 0.044 | 0.548 | -0.060 | | 0.113 |
|  | *Log likelihood (model)* | *-2043.20* | *-1954.94* | | | | | | | | | | |
|  | *AIC* | *4134.40* | *3957.88* | | | | | | | | | | |
|  | *BIC* | *4308.64* | *4132.04* | | | | | | | | | | |
| *Mean= mean coefficients; se= standard error; p= p-value; CI= confidence interval; AIC= Akaike information criterion; BIC= Bayesian information criterion* | | | | | | | | | | | | | |
