# Supplementary material for: Analysis of patients preferences in type 2 diabetes mellitus second-line drug treatment: A discrete choice experiment
Source: PLoS One. 2025 Sep 15;20(9):e0329743. doi: 10.1371/journal.pone.0329743 (PMC12435682; doi:10.1371/journal.pone.0329743)
Supplement: S4 Table — (DOCX) [file pone.0329743.s007.docx]

*S4 Table – CL by BMI categories*

|  |  | **All**  **(N=583)** | | **BMI<25**  **(N=150)** | **BMI 25-29.9**  **(N=356)** | **BMI>=30**  **(N=77)** | | | | | | | | | | | | | | | |
| --- | --- | --- | --- | --- | --- | --- | --- | --- | --- | --- | --- | --- | --- | --- | --- | --- | --- | --- | --- | --- | --- |
| **Attributes** | **Levels** | **Mean** | **se** | **p** | **95% CI** | | **Mean** | **se** | **p** | **95% CI** | | **Mean** | **se** | **p** | **95% CI** | | **Mean** | **se** | **p** | **95% CI** | |
| Risk of  myocardial  infarction | 0 out of 100 patients (0%) | 0.71 | 0.04 | 0.00 | 0.63 | 0.80 | 0.69 | 0.08 | 0.00 | 0.53 | 0.85 | 0.76 | 0.05 | 0.00 | 0.66 | 0.87 | 0.59 | 0.11 | 0.00 | 0.37 | 0.81 |
|  | 2 out of 100 patients (2%) | 0.18 | 0.04 | 0.00 | 0.09 | 0.26 | 0.16 | 0.08 | 0.06 | -0.01 | 0.32 | 0.20 | 0.05 | 0.00 | 0.10 | 0.31 | 0.15 | 0.12 | 0.20 | -0.08 | 0.38 |
|  | 4 out of 100 patients (4%) | -0.24 | 0.04 | 0.00 | -0.33 | -0.16 | -0.22 | 0.08 | 0.01 | -0.38 | -0.05 | -0.28 | 0.06 | 0.00 | -0.39 | -0.16 | -0.20 | 0.12 | 0.09 | -0.43 | 0.03 |
|  | 7 out of 100 patients (7%) | -0.65 | 0.05 | 0.00 | -0.74 | -0.56 | -0.63 | 0.09 | 0.00 | -0.81 | -0.46 | -0.69 | 0.06 | 0.00 | -0.81 | -0.57 | -0.54 | 0.13 | 0.00 | -0.79 | -0.30 |
| Risk of  stroke | 0 out of 100 patients (0%) | 0.55 | 0.04 | 0.00 | 0.47 | 0.63 | 0.47 | 0.08 | 0.00 | 0.30 | 0.63 | 0.62 | 0.05 | 0.00 | 0.52 | 0.72 | 0.44 | 0.12 | 0.00 | 0.22 | 0.67 |
|  | 1 out of 100 patients (1%) | 0.15 | 0.04 | 0.00 | 0.07 | 0.23 | 0.16 | 0.09 | 0.07 | -0.01 | 0.32 | 0.14 | 0.05 | 0.01 | 0.03 | 0.25 | 0.25 | 0.12 | 0.03 | 0.02 | 0.48 |
|  | 2 out of 100 patients (2%) | -0.15 | 0.04 | 0.00 | -0.23 | -0.07 | -0.11 | 0.08 | 0.20 | -0.27 | 0.06 | -0.18 | 0.06 | 0.00 | -0.29 | -0.08 | -0.16 | 0.12 | 0.18 | -0.40 | 0.07 |
|  | 4 out of 100 patients (4%) | -0.55 | 0.05 | 0.00 | -0.64 | -0.46 | -0.52 | 0.09 | 0.00 | -0.70 | -0.34 | -0.58 | 0.06 | 0.00 | -0.69 | -0.46 | -0.53 | 0.13 | 0.00 | -0.78 | -0.29 |
| Risk of  nerve damage | 0 out of 100 patients (0%) | 1.48 | 0.04 | 0.00 | 1.40 | 1.57 | 1.53 | 0.09 | 0.00 | 1.35 | 1.70 | 1.50 | 0.06 | 0.00 | 1.38 | 1.61 | 1.49 | 0.12 | 0.00 | 1.25 | 1.73 |
|  | 5 out of 100 patients (5%) | 0.29 | 0.04 | 0.00 | 0.21 | 0.37 | 0.42 | 0.08 | 0.00 | 0.26 | 0.59 | 0.24 | 0.06 | 0.00 | 0.14 | 0.35 | 0.24 | 0.12 | 0.03 | 0.02 | 0.47 |
|  | 10 out of 100 patients (10%) | -0.19 | 0.04 | 0.00 | -0.28 | -0.10 | -0.26 | 0.09 | 0.00 | -0.44 | -0.09 | -0.18 | 0.06 | 0.00 | -0.29 | -0.08 | -0.11 | 0.12 | 0.36 | -0.35 | 0.13 |
|  | 15 out of 100 patients (15%) | -1.58 | 0.06 | 0.00 | -1.70 | -1.46 | -1.69 | 0.13 | 0.00 | -1.94 | -1.44 | -1.55 | 0.08 | 0.00 | -1.70 | -1.41 | -1.62 | 0.17 | 0.00 | -1.96 | -1.29 |
| Risk of  nausea | 0 out of 100 patients (0%) | 2.45 | 0.06 | 0.00 | 2.34 | 2.56 | 2.75 | 0.13 | 0.00 | 2.50 | 3.00 | 2.45 | 0.07 | 0.00 | 2.31 | 2.59 | 2.30 | 0.14 | 0.00 | 2.02 | 2.59 |
|  | 10 out of 100 patients (10%) | 0.97 | 0.05 | 0.00 | 0.88 | 1.06 | 1.12 | 0.11 | 0.00 | 0.92 | 1.33 | 0.99 | 0.06 | 0.00 | 0.88 | 1.11 | 0.84 | 0.12 | 0.00 | 0.60 | 1.07 |
|  | 30 out of 100 patients (30%) | -1.21 | 0.06 | 0.00 | -1.33 | -1.09 | -1.03 | 0.13 | 0.00 | -1.29 | -0.76 | -1.26 | 0.08 | 0.00 | -1.42 | -1.10 | -1.26 | 0.16 | 0.00 | -1.57 | -0.94 |
|  | 50 out of 100 patients (50%) | -2.21 | 0.08 | 0.00 | -2.37 | -2.05 | -2.85 | 0.23 | 0.00 | -3.30 | -2.39 | -2.18 | 0.10 | 0.00 | -2.39 | -1.98 | -1.88 | 0.18 | 0.00 | -2.25 | -1.52 |
| Risk of severe  hypoglycemia | 0 out of 100 patients (0%) | 0.58 | 0.04 | 0.00 | 0.50 | 0.66 | 0.60 | 0.08 | 0.00 | 0.44 | 0.76 | 0.59 | 0.05 | 0.00 | 0.48 | 0.69 | 0.57 | 0.11 | 0.00 | 0.35 | 0.79 |
|  | 1 out of 100 patients (1%) | 0.11 | 0.04 | 0.01 | 0.03 | 0.20 | 0.14 | 0.08 | 0.08 | -0.02 | 0.30 | 0.12 | 0.05 | 0.03 | 0.01 | 0.22 | 0.05 | 0.11 | 0.69 | -0.18 | 0.27 |
|  | 2 out of 100 patients (2%) | -0.19 | 0.04 | 0.00 | -0.27 | -0.10 | -0.24 | 0.09 | 0.01 | -0.41 | -0.07 | -0.18 | 0.06 | 0.00 | -0.29 | -0.07 | -0.11 | 0.12 | 0.33 | -0.34 | 0.12 |
|  | 4 out of 100 patients (4%) | -0.51 | 0.05 | 0.00 | -0.60 | -0.42 | -0.50 | 0.09 | 0.00 | -0.68 | -0.32 | -0.52 | 0.06 | 0.00 | -0.64 | -0.41 | -0.50 | 0.12 | 0.00 | -0.74 | -0.25 |
| Weight  change | Decrease of -6kg | 0.98 | 0.04 | 0.00 | 0.89 | 1.06 | 0.57 | 0.08 | 0.00 | 0.41 | 0.74 | 1.01 | 0.06 | 0.00 | 0.90 | 1.12 | 1.66 | 0.13 | 0.00 | 1.40 | 1.91 |
|  | Decrease of -2kg | 1.03 | 0.04 | 0.00 | 0.95 | 1.12 | 0.91 | 0.08 | 0.00 | 0.75 | 1.07 | 1.09 | 0.05 | 0.00 | 0.99 | 1.20 | 1.14 | 0.12 | 0.00 | 0.91 | 1.38 |
|  | Increase of +2kg | -0.45 | 0.05 | 0.00 | -0.54 | -0.35 | -0.22 | 0.09 | 0.02 | -0.39 | -0.04 | -0.47 | 0.06 | 0.00 | -0.58 | -0.35 | -0.78 | 0.14 | 0.00 | -1.06 | -0.50 |
|  | Increase of +6kg | -1.56 | 0.06 | 0.00 | -1.68 | -1.45 | -1.26 | 0.11 | 0.00 | -1.48 | -1.05 | -1.64 | 0.08 | 0.00 | -1.80 | -1.49 | -2.02 | 0.19 | 0.00 | -2.40 | -1.64 |
| Type and  frequency  of intake | Oral 1x per week | 0.58 | 0.03 | 0.00 | 0.52 | 0.64 | 0.65 | 0.06 | 0.00 | 0.53 | 0.77 | 0.56 | 0.04 | 0.00 | 0.49 | 0.64 | 0.55 | 0.09 | 0.00 | 0.38 | 0.72 |
|  | Oral 7 times a week | -0.18 | 0.03 | 0.00 | -0.24 | -0.12 | -0.18 | 0.06 | 0.00 | -0.31 | -0.06 | -0.16 | 0.04 | 0.00 | -0.24 | -0.08 | -0.30 | 0.09 | 0.00 | -0.48 | -0.13 |
|  | Injection 1x a week | 0.35 | 0.03 | 0.00 | 0.29 | 0.41 | 0.35 | 0.06 | 0.00 | 0.23 | 0.47 | 0.35 | 0.04 | 0.00 | 0.27 | 0.42 | 0.38 | 0.08 | 0.00 | 0.21 | 0.54 |
|  | Injection 7x a week | -0.75 | 0.03 | 0.00 | -0.82 | -0.68 | -0.82 | 0.07 | 0.00 | -0.96 | -0.68 | -0.75 | 0.05 | 0.00 | -0.84 | -0.66 | -0.62 | 0.09 | 0.00 | -0.80 | -0.44 |
| Schedule of  intake | Independent of meals in the morning | 0.05 | 0.03 | 0.09 | -0.01 | 0.11 | 0.04 | 0.06 | 0.55 | -0.09 | 0.16 | 0.05 | 0.04 | 0.24 | -0.03 | 0.13 | 0.08 | 0.08 | 0.34 | -0.09 | 0.25 |
|  | Dependent on meals in the morning | -0.11 | 0.03 | 0.00 | -0.18 | -0.05 | -0.16 | 0.06 | 0.01 | -0.29 | -0.03 | -0.10 | 0.04 | 0.01 | -0.18 | -0.03 | -0.05 | 0.09 | 0.53 | -0.22 | 0.11 |
|  | Independent of meals in the evening | 0.04 | 0.03 | 0.23 | -0.02 | 0.10 | 0.14 | 0.06 | 0.02 | 0.02 | 0.26 | 0.01 | 0.04 | 0.72 | -0.06 | 0.09 | -0.07 | 0.08 | 0.41 | -0.24 | 0.10 |
|  | Dependent on meals in the evening | 0.03 | 0.03 | 0.41 | -0.03 | 0.09 | -0.02 | 0.06 | 0.75 | -0.14 | 0.10 | 0.04 | 0.04 | 0.27 | -0.03 | 0.12 | 0.04 | 0.09 | 0.63 | -0.13 | 0.21 |
|  | *Log likelihood (model)* | *-4023.98* | | *-1003.81* | *-2424.69* | *-537.28* | | | | | | | | | | | | | | | |
|  | *AIC* | *8095.96* | | *2055.62* | *4897.39* | *1122.55* | | | | | | | | | | | | | | | |
|  | *BIC* | *8286.80* | | *2213.88* | *5076.39* | *1264.81* | | | | | | | | | | | | | | | |
| *Mean= mean coefficients; se= standard error; p= p-value; CI= confidence interval ; AIC= Akaike information criterion; BIC= Bayesian information criterion* | | | | | | | | | | | | | | | | | | | | | |
