# Supplementary material for: Analysis of patients preferences in type 2 diabetes mellitus second-line drug treatment: A discrete choice experiment
Source: PLoS One. 2025 Sep 15;20(9):e0329743. doi: 10.1371/journal.pone.0329743 (PMC12435682; doi:10.1371/journal.pone.0329743)
Supplement: S5 Table — (DOCX) [file pone.0329743.s008.docx]

*S5 Table – RPL model by populations*

|  |  |  |  | | | | |  | | | | **Population 1**  **(N=292)** |  |  | | |  | | | | | **Population 2**  **(N=291)** |
| --- | --- | --- | --- | --- | --- | --- | --- | --- | --- | --- | --- | --- | --- | --- | --- | --- | --- | --- | --- | --- | --- | --- |
| **Attributes** | **Levels** | **Mean** | **p** | **95% CI** | | | **SD** | | **p (SD)** | | **95% CI (SD)** | | **Mean** | **p** | **95% CI** | | | **SD** | **p (SD)** | | **95% CI (SD)** | |
| Risk of myocardial infarction | 0 out of 100 patients (0%) | 0.84 | 0.00 | 0.69 | 0.99 | | 0.25 | | 0.10 | -0.05 | | 0.55 | 0.92 | 0.00 | 0.78 | 1.07 | | 0.26 | 0.36 | -0.29 | | 0.81 |
|  | 2 out of 100 patients (2%) | 0.22 | 0.00 | 0.08 | 0.36 | | -0.10 | | 0.50 | -0.39 | | 0.19 | 0.21 | 0.00 | 0.07 | 0.36 | | 0.23 | 0.10 | -0.04 | | 0.51 |
|  | 4 out of 100 patients (4%) | -0.21 | 0.00 | -0.35 | -0.06 | | 0.00 | | 0.98 | -0.23 | | 0.23 | -0.36 | 0.00 | -0.51 | -0.22 | | -0.03 | 0.85 | -0.37 | | 0.31 |
|  | 7 out of 100 patients (7%) | -0.86 | 0.00 | -1.02 | -0.69 | | -0.15 | | 0.53 | -0.61 | | 0.31 | -0.78 | 0.00 | -0.94 | -0.61 | | -0.46 | 0.17 | -1.11 | | 0.20 |
| Risk of stroke | 0 out of 100 patients (0%) | 0.64 | 0.00 | 0.50 | 0.78 | | . | | . | . | | . | 0.73 | 0.00 | 0.59 | 0.87 | | . | . | . | | . |
|  | 1 in 100 patients (1%) | 0.16 | 0.03 | 0.02 | 0.30 | | . | | . | . | | . | 0.25 | 0.00 | 0.11 | 0.39 | | . | . | . | | . |
|  | 2 out of 100 patients (2%) | -0.12 | 0.08 | -0.26 | 0.02 | | . | | . | . | | . | -0.29 | 0.00 | -0.43 | -0.15 | | . | . | . | | . |
|  | 4 out of 100 patients (4%) | -0.67 | 0.00 | -0.83 | -0.52 | | . | | . | . | | . | -0.69 | 0.00 | -0.84 | -0.54 | | . | . | . | | . |
| Risk of nerve damage | 0 out of 100 patients (0%) | 1.76 | 0.00 | 1.58 | 1.95 | | -0.46 | | 0.00 | -0.74 | | -0.17 | 2.01 | 0.00 | 1.83 | 2.19 | | -0.26 | 0.08 | -0.54 | | 0.03 |
|  | 5 out of 100 patients (5%) | 0.36 | 0.00 | 0.21 | 0.50 | | -0.16 | | 0.33 | -0.48 | | 0.16 | 0.38 | 0.00 | 0.23 | 0.53 | | -0.34 | 0.02 | -0.63 | | -0.06 |
|  | 10 out of 100 patients (10%) | -0.28 | 0.00 | -0.43 | -0.12 | | -0.24 | | 0.18 | -0.59 | | 0.11 | -0.19 | 0.01 | -0.33 | -0.04 | | -0.19 | 0.16 | -0.46 | | 0.07 |
|  | 15 out of 100 patients (15%) | -1.85 | 0.00 | -2.08 | -1.61 | | 0.86 | | 0.00 | 0.30 | | 1.41 | -2.20 | 0.00 | -2.45 | -1.96 | | 0.79 | 0.00 | 0.35 | | 1.23 |
| Risk of nausea | 0 out of 100 patients (0%) | 3.17 | 0.00 | 2.87 | 3.47 | | 0.49 | | 0.00 | 0.16 | | 0.83 | 3.19 | 0.00 | 2.92 | 3.45 | | 0.63 | 0.00 | 0.35 | | 0.91 |
|  | 10 out of 100 patients (10%) | 1.34 | 0.00 | 1.13 | 1.54 | | 0.25 | | 0.20 | -0.13 | | 0.63 | 1.30 | 0.00 | 1.11 | 1.49 | | 0.42 | 0.00 | 0.17 | | 0.67 |
|  | 30 out of 100 patients (30%) | -1.40 | 0.00 | -1.64 | -1.15 | | -0.74 | | 0.00 | -1.13 | | -0.35 | -1.61 | 0.00 | -1.85 | -1.38 | | 0.67 | 0.00 | 0.38 | | 0.97 |
|  | 50 out of 100 patients (50%) | -3.11 | 0.00 | -3.55 | -2.67 | | 0.00 | | 0.99 | -0.60 | | 0.59 | -2.88 | 0.00 | -3.24 | -2.52 | | -1.73 | 0.00 | -2.24 | | -1.22 |
| Risk of severe hypoglycemia | 0 out of 100 patients (0%) | 0.69 | 0.00 | 0.55 | 0.84 | | . | | . | . | | . | 0.77 | 0.00 | 0.63 | 0.91 | | . | . | . | | . |
|  | 1 in 100 patients (1%) | 0.21 | 0.00 | 0.07 | 0.34 | | . | | . | . | | . | 0.12 | 0.08 | -0.02 | 0.25 | | . | . | . | | . |
|  | 2 out of 100 patients (2%) | -0.26 | 0.00 | -0.41 | -0.12 | | . | | . | . | | . | -0.20 | 0.01 | -0.34 | -0.06 | | . | . | . | | . |
|  | 4 out of 100 patients (4%) | -0.63 | 0.00 | -0.79 | -0.48 | | . | | . | . | | . | -0.69 | 0.00 | -0.85 | -0.54 | | . | . | . | | . |
| Weight change | Decrease of -6kg | 1.05 | 0.00 | 0.86 | 1.24 | | 0.94 | | 0.00 | 0.69 | | 1.19 | 1.46 | 0.00 | 1.27 | 1.65 | | 0.98 | 0.00 | 0.76 | | 1.20 |
|  | Decrease of -2kg | 1.31 | 0.00 | 1.13 | 1.49 | | 0.86 | | 0.00 | 0.61 | | 1.11 | 1.41 | 0.00 | 1.24 | 1.58 | | 0.59 | 0.00 | 0.36 | | 0.81 |
|  | Increase of +2kg | -0.36 | 0.00 | -0.52 | -0.20 | | 0.36 | | 0.05 | 0.00 | | 0.72 | -0.51 | 0.00 | -0.68 | -0.34 | | 0.43 | 0.00 | 0.14 | | 0.72 |
|  | Increase of +6kg | -2.00 | 0.00 | -2.28 | -1.72 | | -2.16 | | 0.00 | -2.74 | | -1.58 | -2.35 | 0.00 | -2.64 | -2.07 | | -2.00 | 0.00 | -2.43 | | -1.56 |
| Type and frequency of intake | Oral 1x per week | 0.80 | 0.00 | 0.67 | 0.93 | | 0.68 | | 0.00 | 0.53 | | 0.83 | 0.68 | 0.00 | 0.57 | 0.80 | | 0.45 | 0.00 | 0.28 | | 0.61 |
|  | Oral 7 times a week | -0.18 | 0.00 | -0.28 | -0.07 | | 0.23 | | 0.07 | -0.02 | | 0.48 | -0.27 | 0.00 | -0.38 | -0.16 | | 0.25 | 0.00 | 0.09 | | 0.42 |
|  | Injection 1x a week | 0.49 | 0.00 | 0.38 | 0.60 | | 0.18 | | 0.24 | -0.12 | | 0.48 | 0.44 | 0.00 | 0.33 | 0.55 | | 0.27 | 0.01 | 0.08 | | 0.46 |
|  | Injection 7x a week | -1.11 | 0.00 | -1.27 | -0.95 | | -1.09 | | 0.00 | -1.52 | | -0.66 | -0.85 | 0.00 | -0.99 | -0.71 | | -0.97 | 0.00 | -1.24 | | -0.69 |
| Schedule of intake | Independent of meals in the morning | 0.07 | 0.17 | -0.03 | 0.18 | | -0.04 | | 0.85 | -0.42 | | 0.35 | 0.04 | 0.41 | -0.06 | 0.15 | | . | . | . | | . |
|  | Dependent on meals in the morning | -0.10 | 0.06 | -0.21 | 0.00 | | 0.03 | | 0.87 | -0.36 | | 0.43 | -0.17 | 0.00 | -0.27 | -0.06 | | . | . | . | | . |
|  | Independent of meals in the evening | 0.03 | 0.60 | -0.07 | 0.13 | | -0.04 | | 0.79 | -0.34 | | 0.26 | 0.07 | 0.17 | -0.03 | 0.17 | | . | . | . | | . |
|  | Dependent on meals in the evening | 0.00 | 0.98 | -0.10 | 0.10 | | 0.05 | | 0.86 | -0.49 | | 0.58 | 0.05 | 0.31 | -0.05 | 0.15 | | . | . | . | | . |
|  | *Log likelihood (model)* | *1963.12* | | | | *-1882.74* | | | | | | | | | | | | | | | | |
|  | *Degrees of freedom* | *42* | | | | *39* | | | | | | | | | | | | | | | | |
|  | *AIC* | *4010.25* | | | | *3843.48* | | | | | | | | | | | | | | | | |
|  | *BIC* | *4315.18* | | | | *4126.49* | | | | | | | | | | | | | | | | |
| *Mean= mean coefficients; p= p-value; SD= standard deviations; CI= confidence interval ; AIC= Akaike information criterion; BIC= Bayesian information criterion*  *The sign of the estimated standard deviations is irrelevant: interpret them as being positive.* | | | | | | | | | | | | | | | | | | | | | | |
