# Supplementary material for: Analysis of patients preferences in type 2 diabetes mellitus second-line drug treatment: A discrete choice experiment
Source: PLoS One. 2025 Sep 15;20(9):e0329743. doi: 10.1371/journal.pone.0329743 (PMC12435682; doi:10.1371/journal.pone.0329743)
Supplement: Attachment — Submitted filename: PLOSONE_DCE_Type2diabetes_2ndlinedrugs_20250317.docx [file pone.0329743.s009.docx]

PONE-D-25-09312

Analysis of Patients Preferences in Type 2 Diabetes Mellitus Second-Line Drug Treatment: A Discrete Choice Experiment.

Thank you for the opportunity to review this paper. Please see my comments below:

**General comments**

1. Regarding funding: I note the research was funded by the pharmaceutical company Novo Nordisk Pharma GmbH. Please confirm that they had no input into the attributes/levels and analysis of the DCE.

Abstract

2. If the word count allows consider adding for type 2 diabetes at the end of the last sentence in the background: This study aimed to elicit preferences for second-line drug treatments *for type 2 diabetes.*

**Introduction**

3. Page 5, lines 57-59: The recent NVL for T2D emphasizes the importance of Shared Decision-Making (SDM), which has become the preferred model of interactions between healthcare providers and patients.

Please add a reference to this sentence.

**Materials and Methods**

4. How were the 24 attributes identified in the literature reduced to 6?

On page 7, lines 120-2, there is some information concerning the following attributes being removed: “Some attributes, such as urinary tract infection, genital infection, risk of cardiovascular death, and hospitalization due to heart failure” However, this does not account for the processes of removal for the others? Please provide additional information about the processes used

5. Page 8, lines 138-140, “Attribute levels were derived from literature and clinical studies, reflecting the range of clinical outcomes.”

Please provide references for the literature and clinical studies.

6. Why was cost not considered an attribute? I’m assuming this varies considerably based on the type of drug, frequency of dosing, and mode of delivery.

*Experimental design*

7. Page 8 lines 144-6, “In addition to these tasks, respondents answered one dominance test (this task was not part of the experimental design) to assess the internal validity of their choices [38].”

For the ease of the reader, I suggest a short sentence explaining the purpose of a dominance test.

8. Did you pilot-test the survey or complete any think-aloud or cognitive interviews? Some of the text in the S2 Table – Descriptive Framework is heavy with medical terminology. I’m curious how easily understood it would have been. Did you use any methods to manage this issue?

*Survey instrument*

9. Page 9, line 164, the paragraph stating: “Respondents first underwent an initial screening to confirm eligibility, which included questions about their consent, T2D diagnosis, current treatment, age, and German language proficiency. The web-based survey began with an introduction and explanations of second line drug treatment for T2D. After providing detailed explanations of the attributes and levels used in the DCE, respondents answered questions about personal experiences, such as nerve damage or nausea.”

Perhaps providing a copy of the survey instrument would help alleviate concerns around language and how easily understood the text/terms were for a medical condition and its health side-effects?

10. On page 10, lines 172-3, you state that the survey concluded with an evaluation of the questionnaire. What insights does that provide?

*Study Population and Data Collection*

11. Data was collected from T2D patients in Germany, with potential participants identified through a

service provider.

Please provide additional details regarding how patients were identified. What service provider? Local Hospital, medical centre?

*Statistical analysis*

12. In my opinion several aspects of analysis are missing:
    a. The functional form, were all attributes linear, or were other distributions tested and used?
    b. Effects coding is discussed in the results but should be stated here
    c. Were all parameters specified as random or non-random what was the process involved in this? How many parameters were random and non-random, and what were the distributions?
    d. What socio-demographic characteristics were hypothesised a priori to have the potential of an interaction, e.g. gender, type of medication 1^st^ or 2^nd^ line, sex, BMI etc
    e. What checklist was used to report the DCE? i.e. ESTIMATE, DIRECT please confirm and add the completed checklist as a table
    f. Did you consider calculating Marginal rates of substitution to value trade-offs? If not, why not? I suggest doing so as another way to value the trade-offs

**Results**

*Conditional Logit Model*

13. I’m curious as to why both CL & RPL results are reported.

The RPL model's model fit is better, suggesting a better model fit, so why not report RPL results only?

*Random Parameter Logit Model*

14. Other than sub-group analysis for BMI did you consider any other sub-group analysis?

For example, regarding the respondents wanting to avoid an increased “risk of nausea” or “risk of nerve damage” was the reasoning for this explored? Did respondents have prior experience of nerve damage or nausea (i.e. chemotherapy, nausea during pregnancy etc)

**Discussion section**

15. Page 22 line 377 states: “Similar trends have been reported in other studies on T2D treatment preferences”.

Please provide references.

**Tables/Figures and Supplementary files**

1. The S1 PRISMA figure should be as per the PRISMA guideline versions <https://www.prisma-statement.org/prisma-2020-flow-diagram> please update.
2. S1 Table—Attribute list: The first and second columns state n=14 and n=6; however, 13 attributes are listed in column 1 and 7 in column 2. Please review and revise.

The attribute named: “Frequency os use (dosage)“ please update to: “*Frequency of use (dosage).“*

3. S2 Figure – Decision model

In the blue box containing the text: “minor benefits expected reduces riks” update to “minor benefits expected reduces risks”

It is not clear to me how this figure relates to the text about the decision model and experimental design on pages 8-9. Is there a way to make this clearer, perhaps labelling the attributes at the bottom, i.e. what T2D and treatment consequences refer to which attributes in the text?

4. S3 Figure—Choice Task, example. This should be in English for the benefit of readers.
5. Figure 1: Coefficients of the CL in 95% CI and relative importance appear to be missing in the text. Please add.
6. Table 1: Overview of current T2D treatment and sociodemographic data

Population 2 adds up to 100.1% please review and correct so the population 2 % totals 100%.

Similarly, for gender & marital status, these totals do not equal 100% review and correct. Check all totals as 100% and update as required.

7. Table 2: Conditional logit model

The risk of severe hypoglycaemia results at 1% is unusual (p-value 0.006) given all the other levels of this attribute are stat significant at < 0.001. Is this result correct? Consider rechecking data and effects coding. The LLR and AIC values are large. Do you know why?

8. Consider Tables 2-3 (CL and RPL model results) and S3-5 Tables if there is another way to present the results as there is a significant amount of numbers and perhaps a figure or graph may represent the strength of preference more clearly?
