# Supplementary material for: Analysis of patients preferences in type 2 diabetes mellitus second-line drug treatment: A discrete choice experiment
Source: PLoS One. 2025 Sep 15;20(9):e0329743. doi: 10.1371/journal.pone.0329743 (PMC12435682; doi:10.1371/journal.pone.0329743)
Supplement: Attachment — Submitted filename: 250611_Response to Reviewers_PONE-D-25-09312.docx [file pone.0329743.s011.docx]

Ann-Kathrin Fischer M.Sc., Researcher, Hochschule Neubrandenburg, Gesellschaft für empirische Beratung mbH, Germany

Hidetaka Hamasaki
Academic Editor, *PLOS ONE*

**Revision of Manuscript:** *PONE-D-25-09312*

Dear Dr. Hamasaki, dear Reviewers,

We sincerely thank you for the opportunity to revise and resubmit our manuscript. We are grateful for the constructive and thoughtful feedback provided by the reviewers and the Academic Editor. Your comments have been highly valuable in improving the clarity, methodological transparency, and overall quality of the manuscript.

In the following, we provide a detailed, point-by-point response to each reviewer comment. Where applicable, we have indicated the corresponding changes made to the manuscript and included relevant text excerpts to facilitate verification.

Sincerely,

Ann-Kathrin Fischer

On behalf of the co-authors

1. Reviewer #1

| Comments | Answer |
| --- | --- |
| We sincerely thank Reviewer 1 for the careful and constructive review of our manuscript. Your comments were very helpful and showed great attention to methodological details. We especially appreciated your suggestions on how to improve the analysis section, the decision model, and the clarity of figures and tables. Based on your feedback, we revised several parts of the manuscript to make our methods and results more transparent and easier to follow. In the following, we respond to each of your comments in detail and describe the changes we made in response. All changes have been marked in the revised manuscript. | |
| Reviewer #1: A solid paper that could be enhanced by considering the analysis section, sub groups and the use of MRS. In my opinion it would have been useful to have collected a cost attribute to enable calculation of willingness to pay. | We sincerely thank the reviewers for their thoughtful and constructive comments on our manuscript. Their suggestions have significantly contributed to improving the clarity and overall quality of our work. We have carefully addressed all points raised and revised the manuscript accordingly. In the following, we provide a detailed, point-by-point response to each comment. |
| General Comments:   1. Regarding funding: I note the research was funded by the pharmaceutical company Novo Nordisk Pharma GmbH. Please confirm that they had no input into the attributes/levels and analysis of the DCE. | We confirm that Novo Nordisk Pharma GmbH, while providing financial support, had no influence on the selection of attributes and levels, nor on the design, conduct, or analysis of the Discrete Choice Experiment. |
| Abstract:   2. If the word count allows consider adding for type 2 diabetes at the end of the last sentence in the background: This study aimed to elicit preferences for second-line drug treatments for type 2 diabetes. | The abbreviation “T2D” was added at the end of the sentence to ensure consistency with the previous use of the term and to remain within the word count limit.  “This study aimed to elicit preferences for second-line drug treatments for T2D.” |
| Introduction:   3. Page 5, lines 57-59: The recent NVL for T2D emphasizes the importance of Shared Decision-Making (SDM), which has become the preferred model of interactions between healthcare providers and patients.   Please add a reference to this sentence. | A reference to the National Care Guideline (NVL) for T2D has been added to support the statement on the relevance of Shared Decision-Making. |
| Materials and Methods:   4. How were the 24 attributes identified in the literature reduced to 6?   On page 7, lines 120-2, there is some information concerning the following attributes being removed: “Some attributes, such as urinary tract infection, genital infection, risk of cardiovascular death, and hospitalization due to heart failure” However, this does not account for the processes of removal for the others? Please provide additional information about the processes used | The initial 24 attributes were presented and discussed in detail during the qualitative pilot interviews. Each participant evaluated the attributes individually and then ranked them using a structured card-sorting exercise. Attributes were first categorized as important or not important, and the most important were ranked to encourage explicit trade-offs. A qualitative content analysis was performed to assess the perceived relevance of each attribute. These results were compared with the rankings to identify those attributes most relevant for decision-making. We have revised the section on qualitative interviews accordingly to clarify the process used to reduce the attributes to a final set of six.  „Each participant was asked to divide the attributes into “important” and “not important,” and then to rank the most important ones. This enabled structured prioritization and required participants to make clear trade-off decisions.  A qualitative content analysis was conducted to evaluate the perceived relevance of each attribute. The findings from this analysis were triangulated with the individual rankings to identify which attributes were considered most decision relevant.” |
| Materials and Methods:   5. Page 8, lines 138-140, “Attribute levels were derived from literature and clinical studies, reflecting the range of clinical outcomes.” | No changes were made to this sentence, as no specific comment or request for revision was provided. |
| Materials and Methods:   6. Why was cost not considered an attribute? I’m assuming this varies considerably based on the type of drug, frequency of dosing, and mode of delivery. | Cost was not included as an attribute because the aim of the study was not to estimate willingness to pay (WTP), but rather to explore patients’ preferences regarding the clinical and experiential trade-offs between effectiveness, side effects, and treatment administration. Including cost could have introduced interpretive bias by shifting respondents’ attention toward monetary considerations, especially given the variability in individual out-of-pocket expenses in publicly funded healthcare systems. We therefore focused on attributes that directly reflect treatment-related benefits and risks from the patient perspective. |
| Experimental Design:   7. Page 8 lines 144-6, “In addition to these tasks, respondents answered one dominance test (this task was not part of the experimental design) to assess the internal validity of their choices [38].”   For the ease of the reader, I suggest a short sentence explaining the purpose of a dominance test. | A brief explanation of the purpose of the dominance test has been added to clarify that it is used to assess whether respondents make consistent choices when one alternative clearly dominates the others.  “In the dominance test, one alternative clearly outperforms the others on all attributes, allowing researchers to check for consistent and attentive responding.” |
| Experimental Design:   8. Did you pilot-test the survey or complete any think-aloud or cognitive interviews? Some of the text in the S2 Table – Descriptive Framework is heavy with medical terminology. I’m curious how easily understood it would have been. Did you use any methods to manage this issue? | While this aspect was briefly mentioned in the Discussion section, we agree that it deserves more prominence in the main text. We have therefore added a statement to the “Survey Instrument” section clarifying that pretest interviews were conducted to ensure comprehensibility and usability of the survey instrument, particularly regarding medical terminology used in the attribute descriptions.  “To ensure comprehensibility and usability, particularly with regard to medical terminology used in the attribute descriptions, pretest interviews using the Think-Aloud method were conducted with five individuals diagnosed with T2D. These interviews helped identify unclear or overly technical phrasing and informed subsequent refinements of the survey instrument.“ |
| Survey Instrument:   9. Page 9, line 164, the paragraph stating: “Respondents first underwent an initial screening to confirm eligibility, which included questions about their consent, T2D diagnosis, current treatment, age, and German language proficiency. The web-based survey began with an introduction and explanations of second line drug treatment for T2D. After providing detailed explanations of the attributes and levels used in the DCE, respondents answered questions about personal experiences, such as nerve damage or nausea.”   Perhaps providing a copy of the survey instrument would help alleviate concerns around language and how easily understood the text/terms were for a medical condition and its health side-effects? | Thank you for your suggestion. To ensure full transparency regarding the linguistic and conceptual comprehensibility of the survey, we have provided the complete survey instrument as supplementary material for editorial and peer review purposes.  Specifically, we have included:   - The original German version of the survey, which was rigorously pretested for clarity, comprehensibility, and content validity among individuals with type 2 diabetes prior to study launch. - An English translation of the survey generated using Microsoft Word’s built-in translation feature. While not a formally certified translation, it allows reviewers to navigate the content more easily while maintaining a direct reference to the original German source text. |
| Survey Instrument:   10. On page 10, lines 172-3, you state that the survey concluded with an evaluation of the questionnaire. What insights does that provide? | The evaluation provided insights into respondents’ perceived comprehensibility and acceptance of the questionnaire. A large majority (81.13%) found the questionnaire “easy” to complete, while 8.23% rated it as “very easy” and only 10.12% selected a neutral response (“teils-teils”); very few participants found it “difficult” (0.51%). Regarding future participation, 60.28% indicated they would “likely” fill out a similar questionnaire again, 22.80% answered “very likely,” and only a small minority expressed reluctance (2.59%). These results suggest high cognitive accessibility and broad acceptance of the survey instrument. |
| Study Population and Data Collection   11. Data was collected from T2D patients in Germany, with potential participants identified through a service provider.   Please provide additional details regarding how patients were identified. What service provider? Local Hospital, medical centre? | Participants were recruited through a professional healthcare market research service provider. The recruitment strategy included multiple channels such as panel respondents, individuals from disease-specific self-help groups, and outreach via healthcare-related networks. All participants met predefined eligibility criteria and confirmed a diagnosis of T2D before participation.  “Data was collected from T2D patients in Germany, with potential participants identified through a professional healthcare market research provider using panels, self-help groups, and healthcare-related networks.“ |
| Statistical Analysis:   12. In my opinion several aspects of analysis are missing:     a. The functional form, were all attributes linear, or were other distributions tested and used?     b. Effects coding is discussed in the results but should be stated here     c. Were all parameters specified as random or non-random what was the process involved in this? How many parameters were random and non-random, and what were the distributions?     d. What socio-demographic characteristics were hypothesised a priori to have the potential of an interaction, e.g. gender, type of medication 1st or 2nd line, sex, BMI etc     e. What checklist was used to report the DCE? i.e. ESTIMATE, DIRECT please confirm and add the completed checklist as a table     f. Did you consider calculating Marginal rates of substitution to value trade-offs? If not, why not? I suggest doing so as another way to value the trade-offs | **a. The functional form, were all attributes linear, or were other distributions tested and used?**  All attributes were specified as categorical variables using effect-coding. This approach allows for the estimation of non-linear effects between attribute levels, and avoids assumptions of linearity across levels. No alternative functional forms were tested. The modeling approach reflects the assumption that preferences differ across distinct attribute levels and allows the analysis of the relative importance of levels and attributes. Our study aimed to capture discrete preferences across different levels. We added following sentence to the text.  “All attributes were modeled as categorical variables using effect coding to account for non-linear differences between levels.”  **b. Effects coding is discussed in the results but should be stated here.**  Yes, thank you very much. We have now added a short description of the coding method to Statistical Analysis section.  “All attributes were modeled as categorical variables using effect coding to account for non-linear differences between levels. This method estimates the impact of each level relative to the overall mean of the attribute, rather than a single reference category, and enables the identification of non-linear preference structures across attribute levels."  Bech, Mickael, and Dorte Gyrd‐Hansen. "Effects coding in discrete choice experiments." Health economics 14.10 (2005): 1079-1083.  **c. Were all parameters specified as random or non-random what was the process involved in this? How many parameters were random and non-random, and what were the distributions?**  Thank you very much! Originally, attributes were specified as random to allow maximum flexibility in capturing preference heterogeneity. In the final RPL model, only the attributes with statistically significant standard deviations were retained as random. The remaining attributes were treated as fixed. It was assumed that all random parameters follow a normal distribution.  “In an initial specification, all attribute-level parameters were modeled as random to account for preference heterogeneity. Based on model fit and statistical significance of estimated standard deviations, only parameters with significant heterogeneity were retained as random in the final model. All random parameters were assumed to follow a normal distribution; remaining parameters were modeled as fixed.”  **d. What socio-demographic characteristics were hypothesised a priori to have the potential of an interaction, e.g. gender, type of medication 1st or 2nd line, sex, BMI etc**  Thank you very much! The analysis focused on estimating main effects to identify and quantify the relative importance of treatment attributes and levels on therapy decisions. Subgroup-specific preference heterogeneity was hypothesized with respect to BMI, based on the assumption that the importance of weight change may differ by weight status.  Interaction analyses for other sociodemographic variables were considered in the study design but not pursued in the analysis. This decision was based on the primary aim to determine generalizable main effects and variability in preferences across attribute levels. The identification of distinct preference patterns across multiple covariates will be addressed in a separate analysis, which is planned as a follow-up. We added following to the manuscript.  “The analysis focused on main effects to determine the relative importance of treatment attributes and levels for therapy decisions. Interaction effects with sociodemographic characteristics were not part of the final model. Hypotheses were formulated for BMI, and related subgroup analyses were conducted. See Supplementary Material S3-S5.”  **e. What checklist was used to report the DCE? i.e. ESTIMATE, DIRECT please confirm and add the completed checklist as a table**  Thank you very much! The study followed guidelines outlined in the ISPOR recommendations.  **f. Did you consider calculating Marginal rates of substitution to value trade-offs? If not, why not? I suggest doing so as another way to value the trade-offs**  Thank you for this suggestion. MRS were not included in the current manuscript, as the main focus was on estimating and interpreting main effects and preference heterogeneity. MRS calculations, e.g. in relation to weight change and risk attributes, are planned for a follow-up analysis. |
| Results:  *Conditional Logit Model:*   13. I’m curious as to why both CL & RPL results are reported.   The RPL model's model fit is better, suggesting a better model fit, so why not report RPL results only? | Thank you very much! The CL model was used as an initial step to assess plausibility, model convergence, and the expected direction of level coefficients. Following your recommendation, we have moved the results of the CL model to the supplementary material. |
| Results:  Random Parameter Logit Model:   14. Other than sub-group analysis for BMI did you consider any other sub-group analysis?   For example, regarding the respondents wanting to avoid an increased “risk of nausea” or “risk of nerve damage” was the reasoning for this explored? Did respondents have prior experience of nerve damage or nausea (i.e. chemotherapy, nausea during pregnancy etc) | **Other than sub-group analysis for BMI did you consider any other sub-group analysis?**  Thank you for this question! Additional subgroup analyses based on sociodemographic or clinical variables were considered during study planning. However, the current mansucript focised on main effects. Further exploration of subgroup differences, e.g. using interaction and latent class models, is planned as part of a follow-up analysis but was beyond the scope of this manuscript.  **For example, regarding the respondents wanting to avoid an increased “risk of nausea” or “risk of nerve damage” was the reasoning for this explored?**  Thank you very much! As described in the manuscript, the attributes and levels were developed based on qualitative interviews. In these interviews, “risk of nausea” and “risk of nerve damage” emerged as relevant. While only a few participants reported personal experience with these effects, many expressed a general desire to avoid such risks.  **Did respondents have prior experience of nerve damage or nausea (i.e. chemotherapy, nausea during pregnancy etc)**  Yes. The survey included items asking whether respondents had ever been diagnosed with nerve damage and whether they experience nausea in their everyday life. Approximately 10% of the total sample reported a diagnosis of nerve damage, and around 20% reported frequent or recurring nausea in daily life. |
| Discussion Section:   15. Page 22 line 377 states: “Similar trends have been reported in other studies on T2D treatment preferences”.   Please provide references. | The corresponding references were already included in the original manuscript but have now been moved closer to the beginning of the paragraph to improve visibility and clarify the link to the statement. No new references were added. |
| Tables Figures and Supplementary Files:   1. The S1 PRISMA figure should be as per the PRISMA guideline versions https://www.prisma-statement.org/prisma-2020-flow-diagram please update. | The PRISMA flow diagram in S1 Fig. has been updated to align with the official PRISMA 2020 guideline version, as suggested. |
| Tables Figures and Supplementary Files:   2. S1 Table—Attribute list: The first and second columns state n=14 and n=6; however, 13 attributes are listed in column 1 and 7 in column 2. Please review and revise.   The attribute named: “Frequency os use (dosage)“ please update to: “Frequency of use (dosage).“ | Thank you for pointing this out. The discrepancies in the reported numbers of attributes have been corrected: column 1 now includes 13 attributes and column 2 includes 7, consistent with the actual listing. The spelling error in “Frequency os use (dosage)” has also been corrected to “Frequency of use (dosage)”. |
| Tables Figures and Supplementary Files:   3. S2 Figure – Decision model   In the blue box containing the text: “minor benefits expected reduces riks” update to “minor benefits expected reduces risks”  It is not clear to me how this figure relates to the text about the decision model and experimental design on pages 8-9. Is there a way to make this clearer, perhaps labelling the attributes at the bottom, i.e. what T2D and treatment consequences refer to which attributes in the text? | The spelling error in the blue box of S2 Figure has been corrected to “minor benefits expected reduces risks.” In addition, we have harmonized the wording used in S2 Figure, S1 Table, and the main text (Decision model section) to ensure consistency. |
| Tables Figures and Supplementary Files:   4. S3 Figure—Choice Task, example. This should be in English for the benefit of readers. | Thank you for this valuable remark. In response, we have replaced the original S3 Figure with an updated version that displays both the original German choice task and its English translation side by side. |
| Tables Figures and Supplementary Files:   5. Figure 1: Coefficients of the CL in 95% CI and relative importance appear to be missing in the text. Please add. | The 95% confidence intervals for each attribute level are already reported in Table 2 and visualized in Figure 1. We have not added them to the main text to maintain readability and avoid overloading the results section with numerical detail. We consider the current presentation sufficient for interpreting the model estimates and their statistical precision. |
| Tables Figures and Supplementary Files:   6. Table 1: Overview of current T2D treatment and sociodemographic data   Population 2 adds up to 100.1% please review and correct so the population 2 % totals 100%.  Similarly, for gender & marital status, these totals do not equal 100% review and correct. Check all totals as 100% and update as required. | We have reviewed the percentages in Table 1. The slight discrepancies in column totals (e.g., 99.9% or 100.1%) are due to rounding to one decimal place. These differences would resolve with more decimal precision and do not reflect any data inconsistency. Therefore, no changes were made to the table. |
| Tables Figures and Supplementary Files:   7. Table 2: Conditional logit model   The risk of severe hypoglycaemia results at 1% is unusual (p-value 0.006) given all the other levels of this attribute are stat significant at < 0.001. Is this result correct? Consider rechecking data and effects coding. The LLR and AIC values are large. Do you know why? | **Table 2: Conditional logit model: The risk of severe hypoglycaemia results at 1% is unusual (p-value 0.006) given all the other levels of this attribute are stat significant at < 0.001. Is this result correct? Consider rechecking data and effects coding. The LLR and AIC values are large. Do you know why?**  Thank you very much! The result ist correct. Effects coding was correctly applied across all levels. The level coefficient for the 1% level of severy hypoglycaemia is close to zero, indicating only a modest impact on choice decisions compared to the other levels of the same attribute. This is reflected in the higher p-value (0.006), though still below conventional significance thresholds.  Regarding the log-likelihood and fit statistics, these values are expected to be large in absolute terms due to the sample size and the number of choice observations per respondent (583 respondents × 12 tasks × 3 alternatives). The LLR and fit criteria should not be interpreted on their own but rather compared across models. The RPL model showed superior fit relative to the CL model, as indicated by a less negative log-likelihood and lower AIC and BIC values. |
| Tables Figures and Supplementary Files:   8. Consider Tables 2-3 (CL and RPL model results) and S3-5 Tables if there is another way to present the results as there is a significant amount of numbers and perhaps a figure or graph may represent the strength of preference more clearly? | The main findings from Tables 2 and 3 are already visualized in the manuscript as figures to support interpretation. The full numerical model results are presented in the supplementary tables to ensure transparency and allow interested readers to explore the detailed coefficients. We consider this combination of figures in the main text and tables in the supplement to provide an appropriate balance between clarity and completeness. |

2. Reviewer #2

| Comments | Answer |
| --- | --- |
| We sincerely thank Reviewer 2 for the kind and constructive review of our manuscript. We appreciate your recognition of the overall quality of the manuscript, as well as your helpful comments on how to further improve its clarity and depth. Your suggestions have helped us to reflect critically on the study design and to improve the explanation of our methodological decisions in the manuscript. In the following, we respond in detail to each of your comments and explain how they have been addressed in the revised version. | |
| Reviewer #2: Dear, I hope this message finds you well. I would like to express my gratitude for the opportunity to review the manuscript titled "Analysis of Patients Preferences in Type 2 Diabetes Mellitus Second-Line Drug Treatment: A Discrete Choice Experiment." Overall, the paper is well-written and presents valuable insights into the topic. However, I have a few comments that I believe could enhance the clarity and depth of the study. | We sincerely thank the reviewers for their thoughtful and constructive comments on our manuscript. Their suggestions have significantly contributed to improving the clarity and overall quality of our work. We have carefully addressed all points raised and revised the manuscript accordingly. In the following, we provide a detailed, point-by-point response to each comment. |
| Firstly, I noticed that in the qualitative study section, there was no consultation with experts in the field of diabetes to complement the literature review. Given that physicians have direct interactions with patients, their insights could be incredibly beneficial to the findings. | We acknowledge the potential value of expert consultation. However, the aim of this study was to exclusively capture the patient perspective. To ensure that the identified attributes genuinely reflect patients’ priorities and experiences, we deliberately focused on qualitative interviews with individuals diagnosed with T2D, without incorporating clinician input at this stage. This approach aligns with the objective of deriving attributes grounded in patient-relevant decision criteria. |
| Secondly, regarding the determination of layers for specific attributes, such as risk of stroke and risk of severe hypoglycemia, the differences between the levels appear to be quite trivial. It would be helpful to clarify the basis for these levels, especially since the differences between them are not tangible. This lack of substantial differentiation may explain why these two attributes showed the least impact in the results, as the distinctions do not seem significant or meaningful. I appreciate your consideration of these points, and I believe addressing them could strengthen the manuscript further. | The reviewer is correct in noting that the level differences for stroke risk and risk of severe hypoglycemia appear small. This was a deliberate choice, as the levels were based on data from clinical studies and represent the actual variation observed in current treatment options. We have slightly expanded the corresponding sentence in the manuscript (section Decision Model) to clarify this rationale.  “Attribute levels were derived from literature and clinical studies, reflecting the range of clinical outcomes. For attributes such as stroke risk and risk of severe hypoglycemia, the level differences were intentionally kept narrow to reflect the actual variation reported in contemporary clinical trials and approved treatment options at the time of study design.“ |
